# Supplementary material for: Causes and consequences of experimental variation in Nicotiana benthamiana transient expression
Source: Nat Commun. 2026 Feb 14;17:2772. doi: 10.1038/s41467-026-69458-1 (PMC13018205; doi:10.1038/s41467-026-69458-1)
Supplement: Supplementary file 1 — Supplementary Information [file 41467_2026_69458_MOESM1_ESM.pdf]

Title: Causes and consequences of experimental variation in *Nicotiana benthamiana* transient expression

Supplementary information

#	Origin	T-DNA description	GenBank	JBEI Part ID
1	pVS1	PCM2:GFP_CaMV35S2:nptII	PX927315	JPUB_021048
2	pVS1	PCH5:CYP76AD1	PX927308	JPUB_026773
3	pVS1	PCH5:DODA	PX927309	JPUB_026771
4	pVS1	PCH5:glycosyltransferase	PX927310	JPUB_026775
5	pVS1	PCH5:RUBY	PX927314	JPUB_026785
6	pVS1	35S:LigA	PX927305	JPUB_018689
7	pVS1	35S:LigB	PX927306	JPUB_018691
8	pVS1	35S:LigC	PX927307	JPUB_018693
9	pVS1	35S:QsuB	PX927311	JPUB_018707
10	pVS1	35S:AroG	PX927304	JPUB_018685
11	pVS1	PCM2:GFP	PX927333	JPUB_026733
12	pVS1	PCM2:mCherry	PX927337	JPUB_026735
13	pVS1	PCM2:GFP_PCM2:mCherry	PX927332	JPUB_026737
14	pVS1	PCM2:mCherry_PCM2:GFP	PX927336	JPUB_026739
15	pVS1	PCM2:GFP_mCherry:PCM2	PX927331	JPUB_026741
16	pVS1	PCM2:mCherry_GFP:PCM2	PX927335	JPUB_026731
17	pVS1	GFP:PCM2_mCherry:PCM2	PX927318	JPUB_026743
18	pVS1	mCherry:PCM2_GFP:PCM2	PX927320	JPUB_026745
19	pVS1	GFP:PCM2_PCM2:mCherry	PX927319	JPUB_026747
20	pVS1	mCherry:PCM2_PCM2:GFP	PX927321	JPUB_026749

21	BBR1	PCM2:mCherry	PX927313	JPUB_026779
22	BBR1	PCM2:GFP	PX927312	JPUB_026777
23	pSa	PCM2:mCherry	PX927317	JPUB_026783
24	pSa	PCM2:GFP	PX927316	JPUB_026781
25	pVS1	PCM2:GFP_REVTOCS_PCM2:mCherry	PX927330	JPUB_026765
26	pVS1	PCM2:mCherry_REVTOCS_PCM2:GFP	PX927334	JPUB_026767
27	pVS1	PCM2:GFP_REVTOCS_mCherry:PCM2	PX927329	JPUB_026769
28	pVS1	PCL2:GFP	PX927326	JPUB_026751
29	pVS1	PCL2:mCherry	PX927327	JPUB_026753
30	pVS1	PCH5:GFP	PX927323	JPUB_026759
31	pVS1	PCH5:mCherry	PX927324	JPUB_026761
32	pVS1	PCL1:mCherry	PX927325	JPUB_026763
33	pVS1	PCM1:mCherry	PX927328	JPUB_026755
34	pVS1	PCH4:mCherry	PX927322	JPUB_026757

Supplementary Table 1. All binary vectors used in this study. T-DNA descriptions are written (left border)-(T-DNA)-(right border). Underscores indicate contiguous expression cassettes that are contained within the same T-DNA. Gene:promoter is used to indicate that the cassette is read in the opposite direction of promoter:gene. All expression cassettes are terminated by T_AtUbq3 with the exception of the nptII cassette in binary vector 1, which is followed by the CaMV 3’ UTR, and the RUBY (vectors 2-5) and PDC (vectors 6-10) binary vectors, which are followed by the CpMV 3’ UTR.

Strain	Description	Reference/Source
E. coli XL1-Blue	Cloning strain of *E. coli*	Agilent
Agrobacterium tumefaciens GV3101:pMP90	Laboratory strain of *A. tumefaciens* C58 that has been disarmed of T-DNA	Intact Genomics
Agrobacterium tumefaciens EHA105	Laboratory strain of *A. tumefaciens* C58 with a hypervirulent pTiBo542 Ti plasmid that has been disarmed of T-DNA	GoldBio

Supplementary Table 2. All bacterial strains used in this study. All plasmids in Supplementary Table 1 were all cloned using *E. coli* and then transformed into *A. tumefaciens* GV3101 for agroinfiltration. Only vector 1 was transformed into *A. tumefaciens* EHA105 for agroinfiltration. *A. tumefaciens* strain GV3101 is resistant to rifampicin and gentamicin, and *A. tumefaciens* strain EHA105 is resistant to rifampicin.

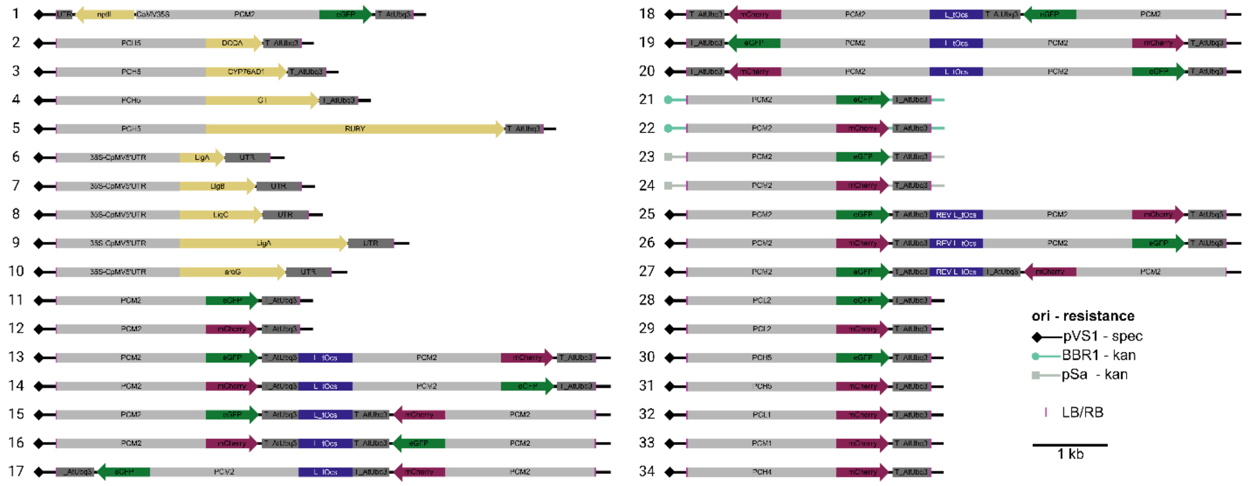

Supplementary Figure 1. Diagram of all binary vectors used in this study. Numbering of plasmids follows the numbering in Supplementary Table 1, *i.e.*, in order of appearance. T-DNAs are written from left border to right border. UTR in vector 1 is the CaMV 3’ UTR, and the UTR in vectors 2-10 is the CpMV 3’ UTR. Created in BioRender. Tang, S. (2025) https://BioRender.com/u99vtsf.

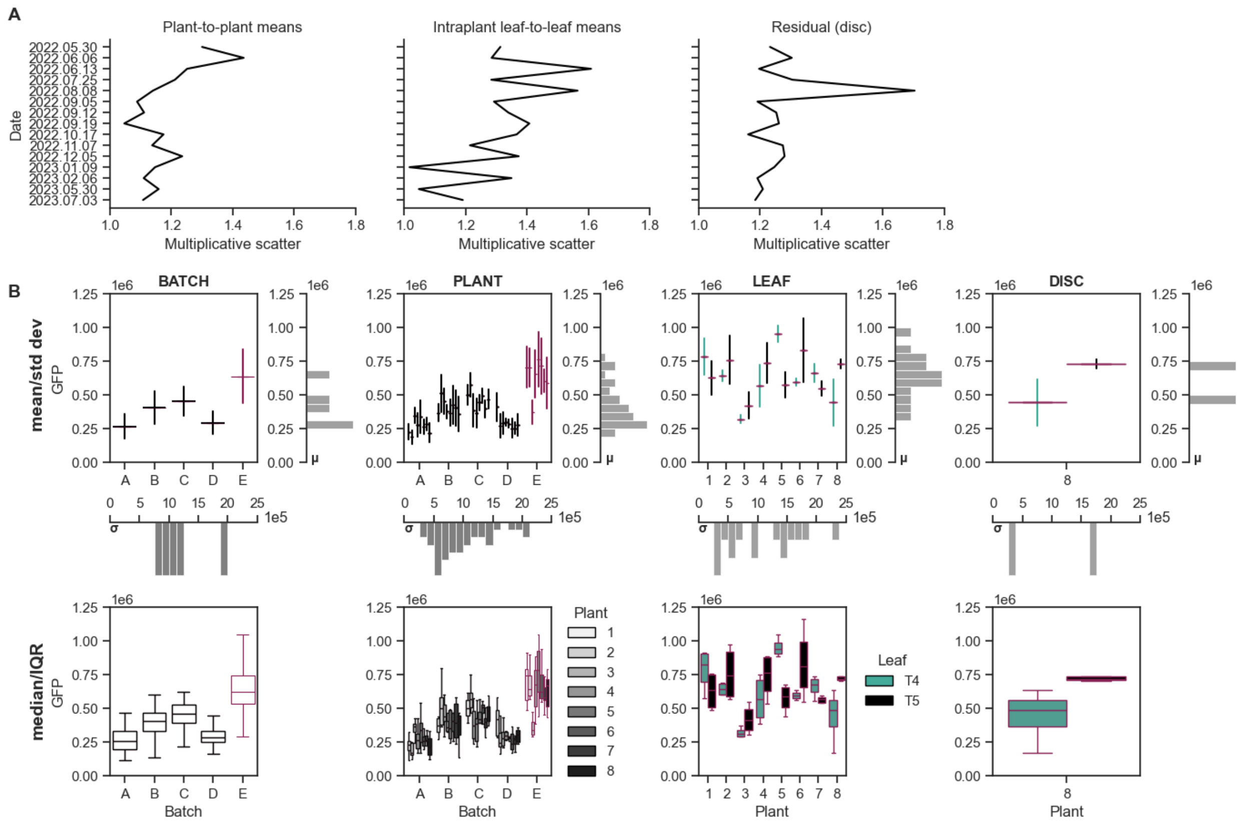

Supplementary Figure 2. Components of the mixed effects model. **A**, multiplicative scatter for each variance component of the mixed effects model fit to 15 independent PCM2:eGFP transient expression experiments (from Fig. 1). Variance components were estimated on the scale to accommodate heteroskedasticity; accordingly, sources of variation are interpreted as multiplicative rather than additive on the original measurement scale. For each component, the estimated standard deviation on the $\log_{10}$ scale ($\sigma_{\log_{10}}$) was calculated, and the back-transformation to the original scale ($10^{\sigma_{\log_{10}}}$), termed multiplicative scatter, was reported. This quantity represents the fold-dispersion around the mean attributable to that component alone, with other components held constant. A value of 1 indicates no additional variability, whereas values >1 indicate increasing multiplicative dispersion (e.g., $\sigma_{\log_{10}} = 0.11 \Rightarrow 10^{0.11} = 1.29$, corresponding to ~29% scatter around the mean). Caterpillar plots show week-specific estimates for between-plant, between-leaf, and residual components. **B**, illustration of the components of the mixed effects model for five selected experimental replicates from Fig. 1. In the “mean/std dev” row, horizontal lines indicate the mean GFP and vertical lines are one standard deviation above and below the mean for a given batch, plant, or leaf. To the right of each subplot is a histogram of GFP means. *The mixed effect model treats the batch-, plant-, and leaf-level GFP means and standard deviations as having random intercepts and random slopes.* In the “median/IQR” row, the center and spread of the data are presented as boxplots (box is the IQR, middle line is the median, whiskers are the minima and maxima excluding outliers, i.e., beyond 1.5 IQR). Source data for this figure is available in the Source Data file.

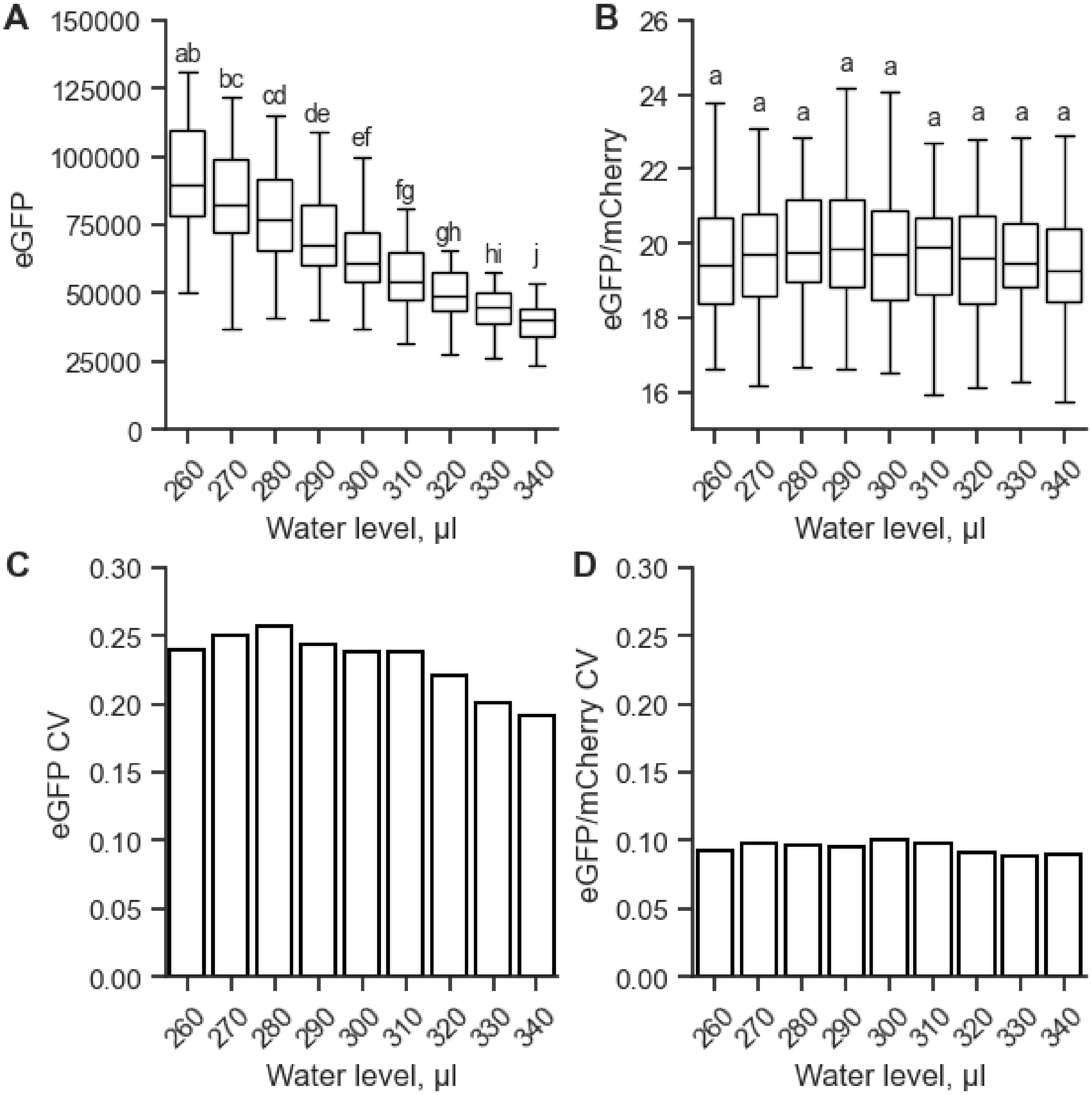

Supplementary Figure 3. Water level (in µL) effects on fluorescence signal strength and variability. 4-week old *N. benthamiana* plants were co-infiltrated using the same two *A. tumefaciens* GV3101 strains, each at an OD of 0.1 and carrying binary vectors with PCM2:eGFP:T_AtUbq3 or PCM2:mCherry:T_AtUbq3 inside the T-DNA.The volume of water was varied inside the 96-well plate used to measure the same leaf discs' fluorescences. Eight plants, two leaves per plant, and four discs per leaf, for a total of n=64 discs. **A**, raw GFP fluorescence. **B**, ratio of GFP fluorescence to mCherry fluorescence. Boxes show the median and IQR, and whiskers show the minima and maxima, excluding outliers (beyond 1.5 IQR). **C**, coefficient of variation of GFP fluorescence. **D**, coefficient of variation of GFP/mCherry fluorescence ratio. An independent 2-sample Student's t-test and a Bonferroni correction were performed between every condition for both raw GFP fluorescence and for GFP/mCherry fluorescence ratio. $p<0.05$ was the cutoff for statistical significance. Shared letters indicate conditions which are not significantly different from one another. Source data for this figure is available in the Source Data file.

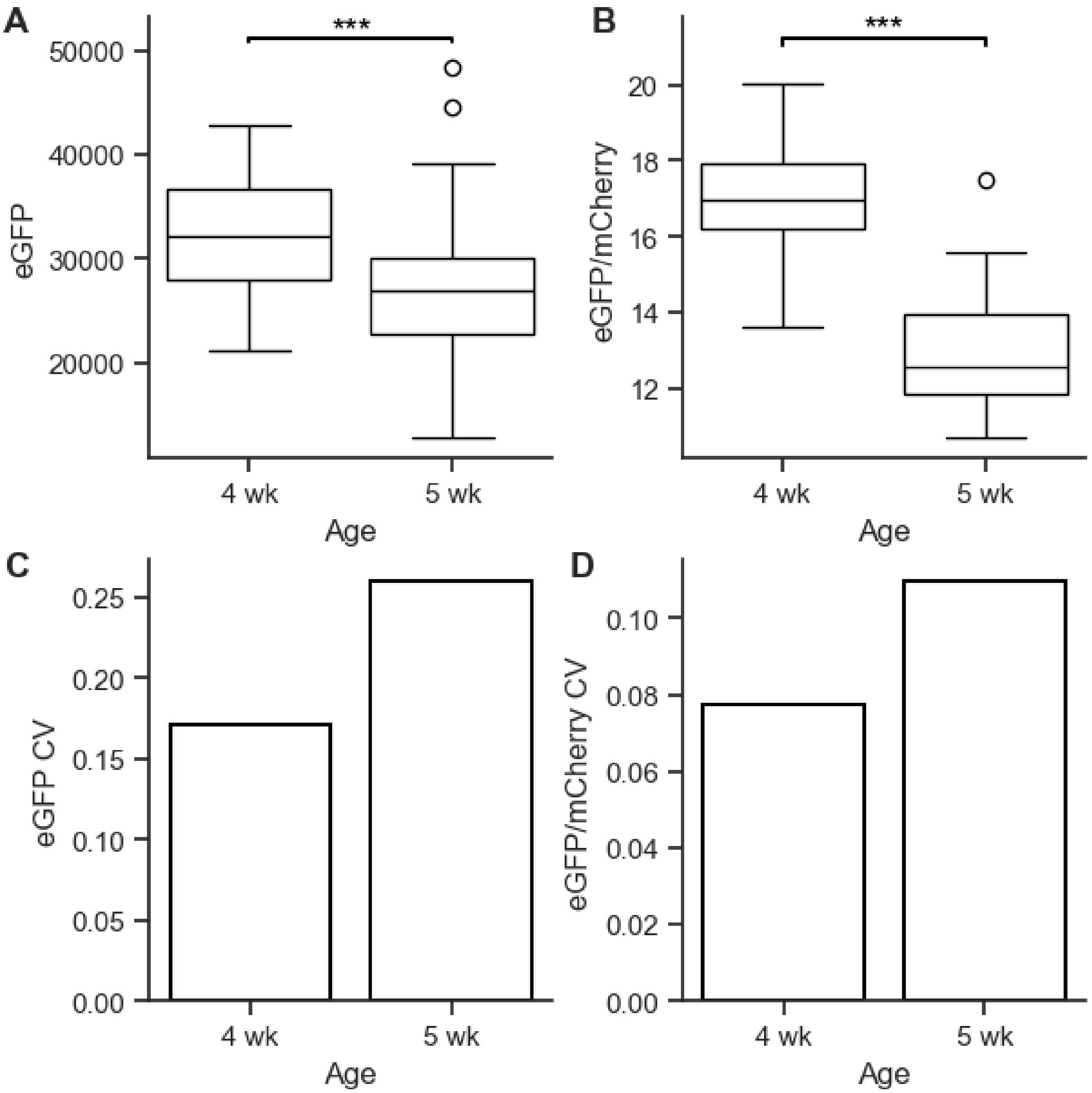

Supplementary Figure 4. Plant age effects on fluorescence signal strength and variability. *N. benthamiana* plants either 4- or 5-weeks old were co-infiltrated using the same two *A. tumefaciens* GV3101 strains, each at an OD of 0.1 and carrying binary vectors with PCM2:eGFP:T_AtUbq3 or PCM2:mCherry:T_AtUbq3 inside the T-DNA. Eight plants per condition, two leaves per plant, and four discs per leaf, for a total of n=64 discs. **A**, raw GFP fluorescence. **B**, ratio of GFP fluorescence to mCherry fluorescence. Boxes show the median and IQR, and whiskers show the minima and maxima, excluding outliers (beyond 1.5 IQR). Circles indicate outliers. **C**, coefficient of variation of GFP fluorescence. **D**, coefficient of variation of GFP/mCherry ratio. An independent 2-sample Student's t-test was performed between the 4- and 5-week plants. *** indicates $p<0.001$. Source data for this figure is available in the Source Data file.

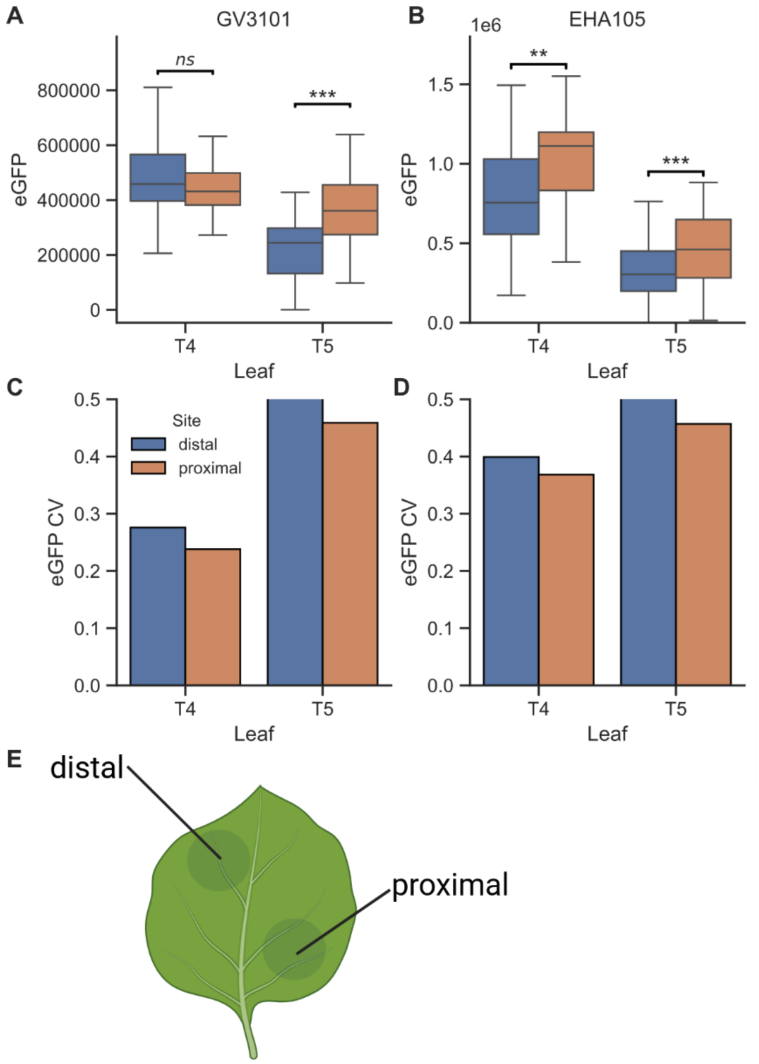

Supplementary Figure 5. Effect of leaf infiltration site on fluorescence signal strength and variability. eGFP measure in leaves T4 and T5 of 4-week old *N. benthamiana* plants infiltrated with either **A**, GV3101, or **B**, EHA105 carrying the same binary vector. Leaves were infiltrated at two sites, distal and proximal to the petiole. Boxes show the median and IQR, and whiskers show the minima and maxima, excluding outliers (beyond 1.5 IQR). eGFP CV of **C**, GV3101 and **D**, EHA105. **E**, illustration of the proximal and distal infiltration sites. Twelve plants per strain, two leaves per plant, two sites per leaf, and four discs per site, for a total of n=96 discs for each unique strain and site combination. A two-tailed Welch's t-test was conducted to compare the distal and proximal sites for each leaf for each strain. Created in BioRender. Tang, S. (2025) https://BioRender.com/4fpcf4p. Source data for this figure is available in the Source Data file.

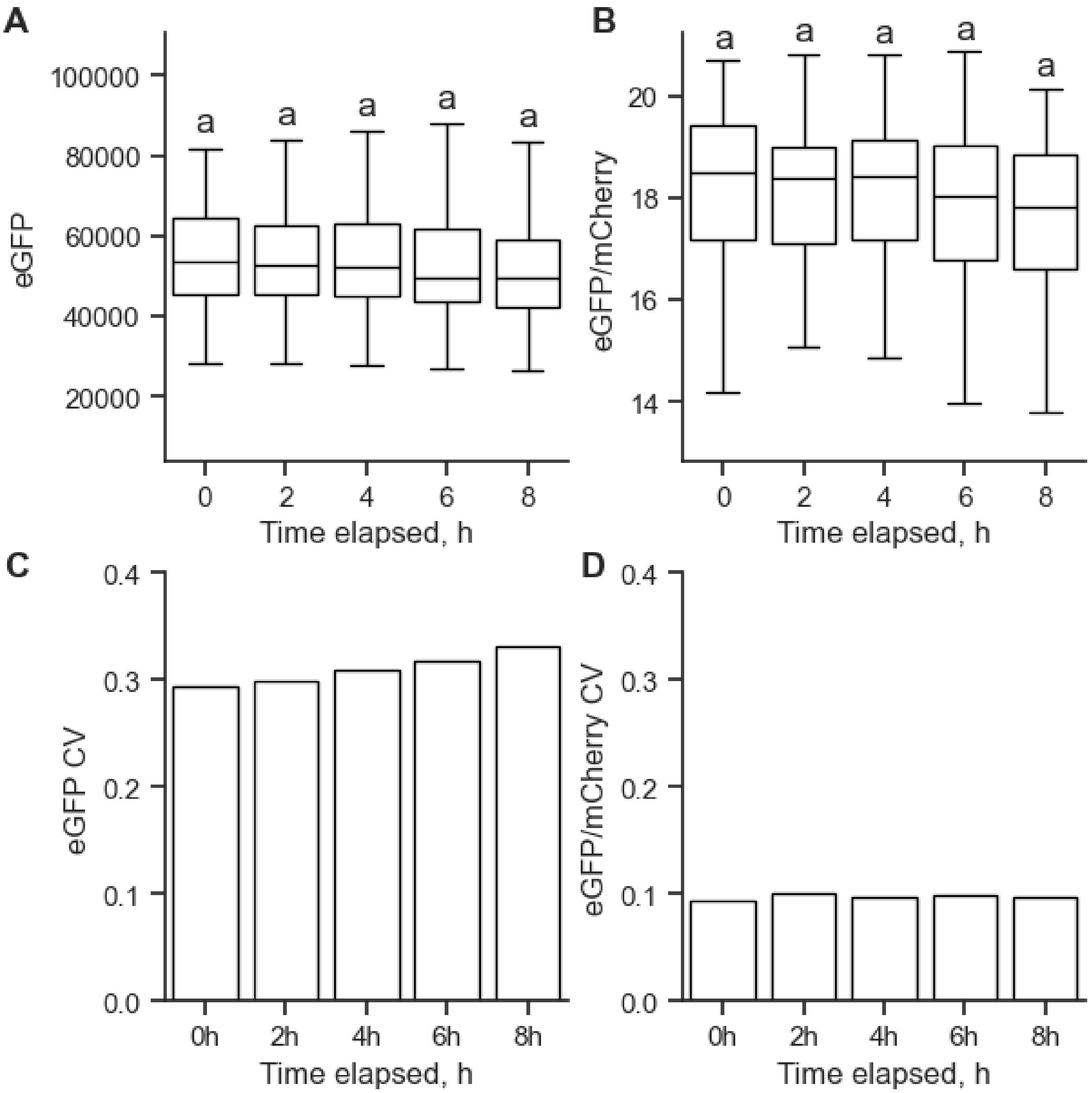

Supplementary Figure 6. Time elapsed between disc collection and measurement effects on fluorescence signal strength and variability. 4-week old *N. benthamiana* plants were co-infiltrated using the same two *A. tumefaciens* GV3101 strains, each at an OD of 0.1 and carrying binary vectors with PCM2:eGFP:T_AtUbq3 or PCM2:mCherry:T_AtUbq3 inside the T-DNA. Six plants, two leaves per plant, and four discs per leaf, for a total of n=48 discs. Discs were collected and then measured on a plate reader every 2 hours thereafter until the end of the experiment. **A**, raw GFP fluorescence. **B**, ratio of GFP fluorescence to mCherry fluorescence. Boxes show the median and IQR, and whiskers show the minima and maxima, excluding outliers (beyond 1.5 IQR). **C**, coefficient of variation of GFP fluorescence. **D**, coefficient of variation of GFP/mCherry ratio. An independent, 2-sample Student's t-test and a Bonferroni correction were performed between every condition for both raw GFP fluorescence and for GFP/mCherry fluorescence ratio. No statistically significant differences were observed with a p-value cutoff of 0.05. Source data for this figure is available in the Source Data file.

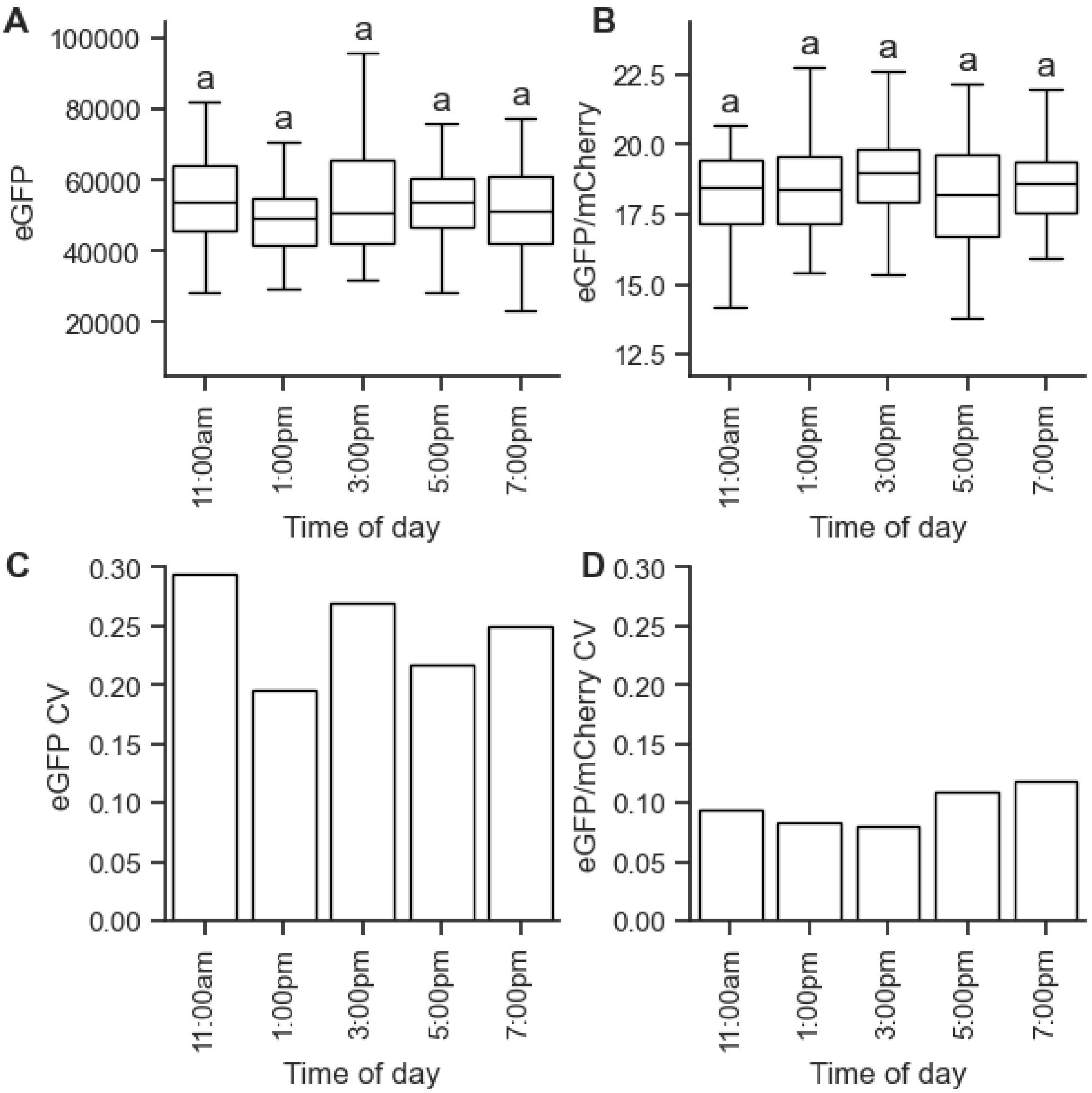

Supplementary Figure 7. Time of day during disc collection and measurement effects on fluorescence signal strength and variability. 4-week old *N. benthamiana* plants were co-infiltrated using the same two *A. tumefaciens* GV3101 strains, each at an OD of 0.1 and carrying binary vectors with PCM2:eGFP:T_AtUbq3 or PCM2:mCherry:T_AtUbq3 inside the T-DNA. Six plants per condition, two leaves per plant, and discs per leaf, for a total of n=48 discs. Each condition represents a group of plants from which discs were collected at the same time (every two hours from 11:00am to 7:00pm). Leaf discs were collected and immediately measured. **A**, raw GFP fluorescence. **B**, ratio of GFP fluorescence to mCherry fluorescence. Boxes show the median and IQR, and whiskers show the minima and maxima, excluding outliers (beyond 1.5 IQR). **C**, coefficient of variation of GFP fluorescence. **D**, coefficient of variation of GFP/mCherry ratio. An independent, 2-sample Student's t-test and a Bonferroni correction were performed between every condition for both raw GFP fluorescence and for GFP/mCherry fluorescence ratio. No statistically significant differences were observed with a p-value cutoff of 0.05. Source data for this figure is available in the Source Data file.

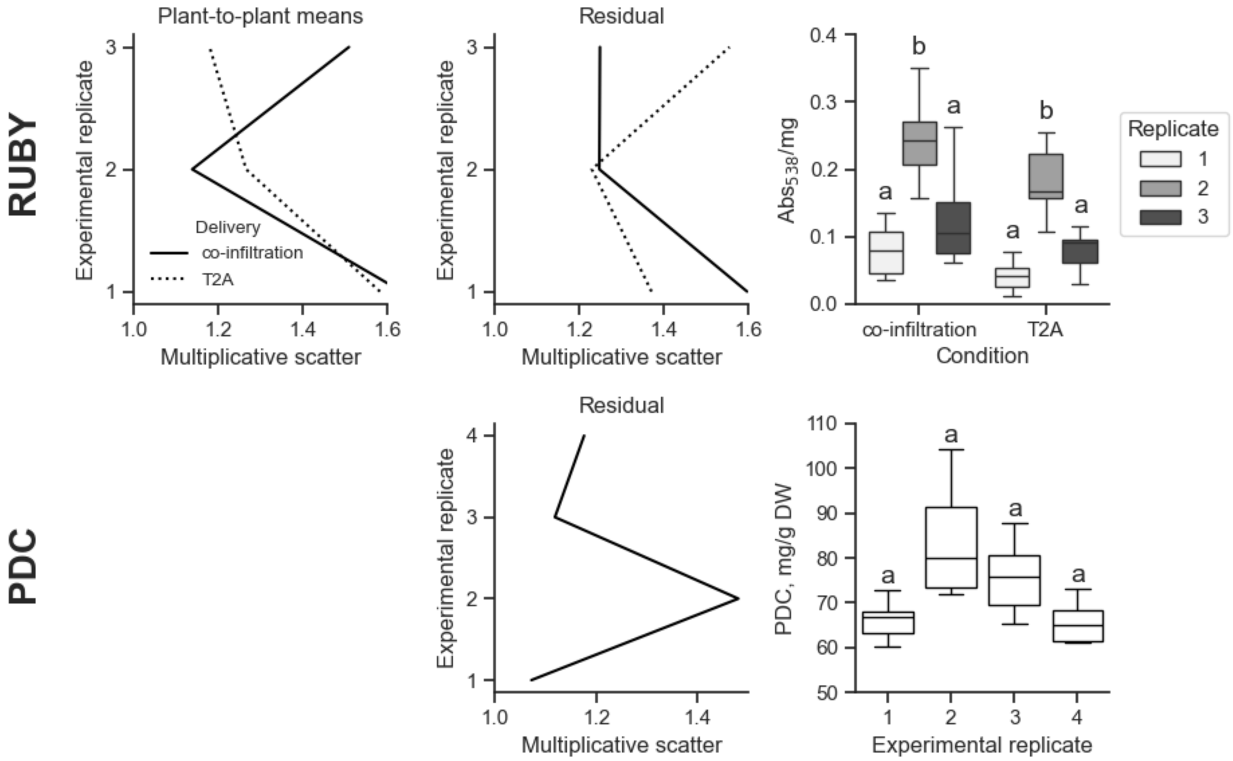

Supplementary Figure 8. Top row, left and center, multiplicative scatters for components of variation in betalain absorbance. Top row, right, absorbance at λ = 538 nm per mg dried tissuefor RUBY reporter agroinfiltrated into *N. benthamiana*. The three enzymes were introduced either on three separate T-DNAs (co-infiltration) or in one T-DNA connected by self-cleaving T2A peptides (T2A). Each independent experimental replicate was conducted with a unique plant batch on separate dates but extracted and measured together. Three experimental replicates of six plants, two leaves per plant, two co-delivery methods per leaf. Bottom row, center, multiplicative scatter for residual variance in 2-pyrone-4,6-dicarboxylic acid (PDC) yields. Bottom row, right, yields of the PDC biosynthetic pathway agroinfiltrated into *N. benthamiana*. Each independent experimental replicate was conducted with a unique plant batch on separate dates but extracted and quantified with HPLC together. Four experimental replicates of six plants, two leaves pooled per plant. Boxes show the median and IQR, and whiskers show the minima and maxima, excluding outliers (beyond 1.5 IQR). An independent, 2-sample Student's t-test and a Bonferroni correction were performed between experimental replicates' λ = 538 nm absorbance or PDC yield per dry weight. $p<0.05$ was the cutoff for statistical significance. Shared letters indicate conditions which are not significantly different from one another. Source data for this figure is available in the Source Data file.

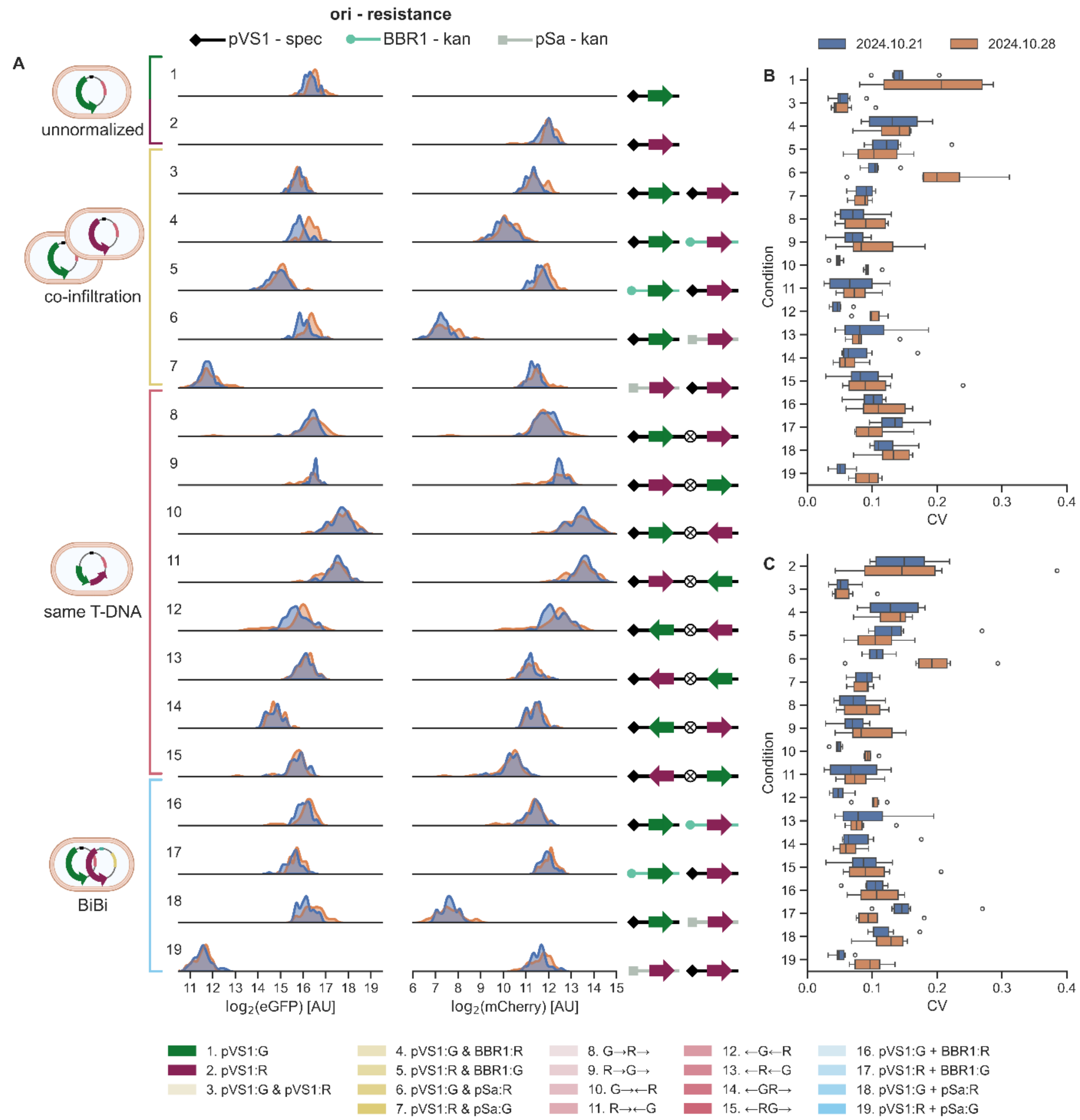

Supplementary Figure 9. Fig 2. data split by experimental replicate. Blue, 2024.10.21. Orange, 2024.10.28. **A)** Left, categories of delivery methods: unnormalized (green or magenta), co-infiltration (yellow), same T-DNA (pink), and BiBi (blue). Center, kernel density estimation plots of eGFP and mCherry fluorescence, n=48 leaf discs per experimental replicate per scheme. Right, cartoons showing the binary vector origin of replication, resistance marker, and orientations of FP expression cassettes in the T-DNA. All binary vector cartoons are read from left to right: ori, left border, T-DNA, right border. Origins of replication are pVS1 (diamond, black), BBR1 (circle, teal), and pSa (square, gray). Circles enclosing an X represent tOcs, a 722bp spacer in between the two expression cassettes. **B)** Plant coefficients of variation (CV), as calculated from the 8 discs per plant, when eGFP is treated as the reporter. All values are eGFP/mCherry CV except for scheme 1, which is GFP CV. **C)** Plant CVs when mCherry is treated as the reporter. All values

are mCherry/eGFP CV except for scheme 2, which is mCherry CV. Normalization scheme IDs match across all subpanels. Blue, experimental replicate conducted on 2024.10.21, and orange, experimental replicate conducted on 2024.10.28. Total OD infiltrated in all schemes is 0.5. ODs of co-infiltrated strains are 0.25 each. Boxes show the median and IQR, and whiskers show the minima and maxima, excluding outliers (beyond 1.5 IQR). Circles indicate outliers. In the legend, "&" indicates co-infiltration, arrows indicate the direction of an expression cassette, "+" indicates BiBi, and GFP is abbreviated to "G" and mCherry to "R". Created in BioRender. Tang, S. (2025) https://BioRender.com/4ila5ts. Source data for this figure is available in the Source Data file.

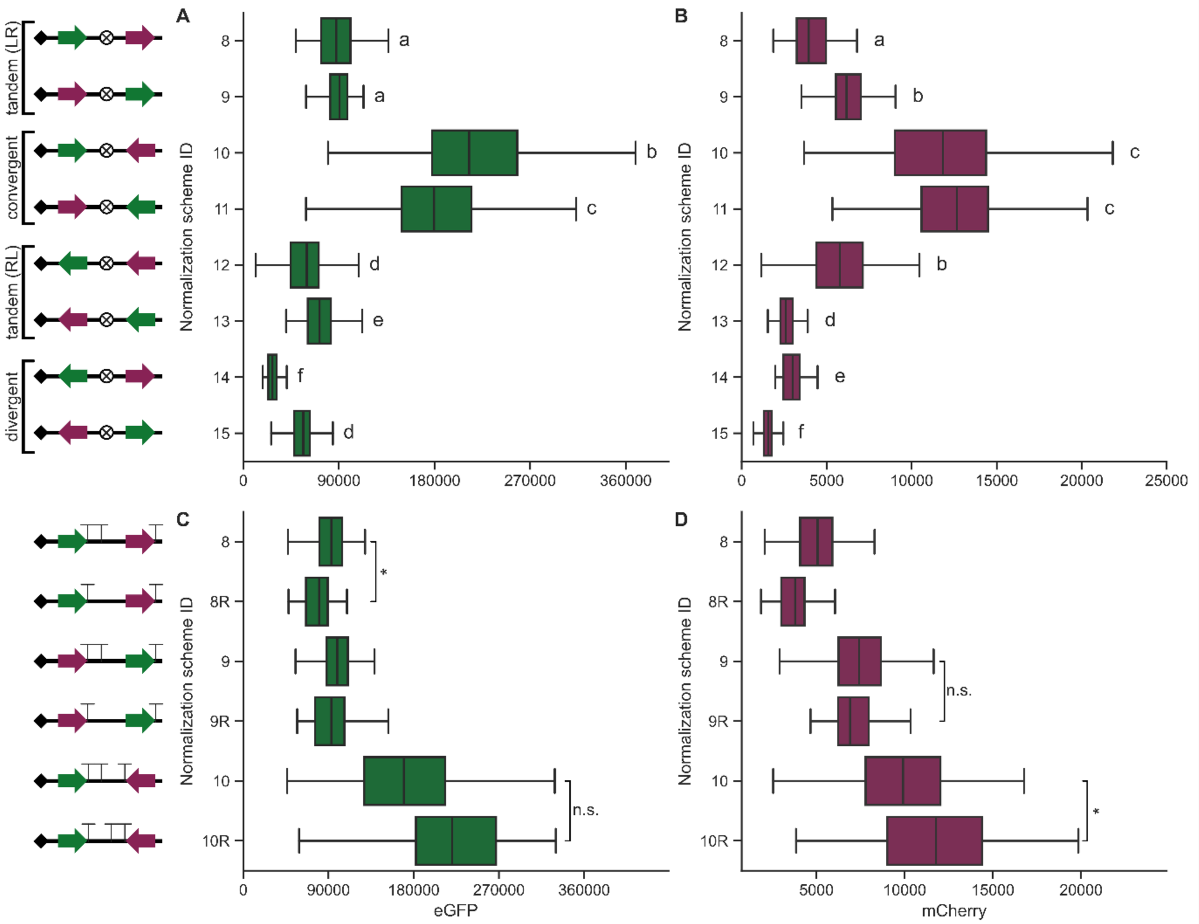

Supplementary Figure 10. Impact of transgene orientation on gene expression. Raw fluorescence signal from **A)** eGFP and **B)** mCherry for schemes 8-15 (two-cassette T-DNA schemes) from Fig. 2A. A two-tailed, independent Student's t-test and Bonferroni correction were conducted between all pairs of schemes, and schemes with significantly different fluorescences are marked with different letters. $p<0.05$ was the cutoff for statistical significance. The label "tandem (LR)" indicates left to right, and "tandem (RL)" indicates right to left. Two experimental replicates were performed for a total of n=96 leaf discs. **C)** eGFP and **D)** mCherry signals for schemes 8, 9, and 10 were also compared to another scheme where the spacer sequence tOcs was reversed (8R, 9R, and 10R). Reversal of tOcs converts a double terminator into a single terminator for schemes 8 and 9 and switches the doubly terminated cassette in scheme 10. Single

terminators are represented as “T”, and double terminators are represented as “TT”. A one-tailed Student’s t-test was conducted to test whether the doubly terminated scheme has higher mean fluorescence than the corresponding singly terminated scheme. * indicates $p<0.05$. Six plants per condition, two leaves per plant, four discs per leaf. Boxes show the median and IQR, and whiskers show the minima and maxima, excluding outliers (beyond 1.5 IQR). Binary vector cartoons are from left to right: ori, left border, cassette 1, 722 bp spacer, cassette 2, right border. Created in BioRender. Tang, S. (2025) https://BioRender.com/w0f87at. Source data for this figure is available in the Source Data file.

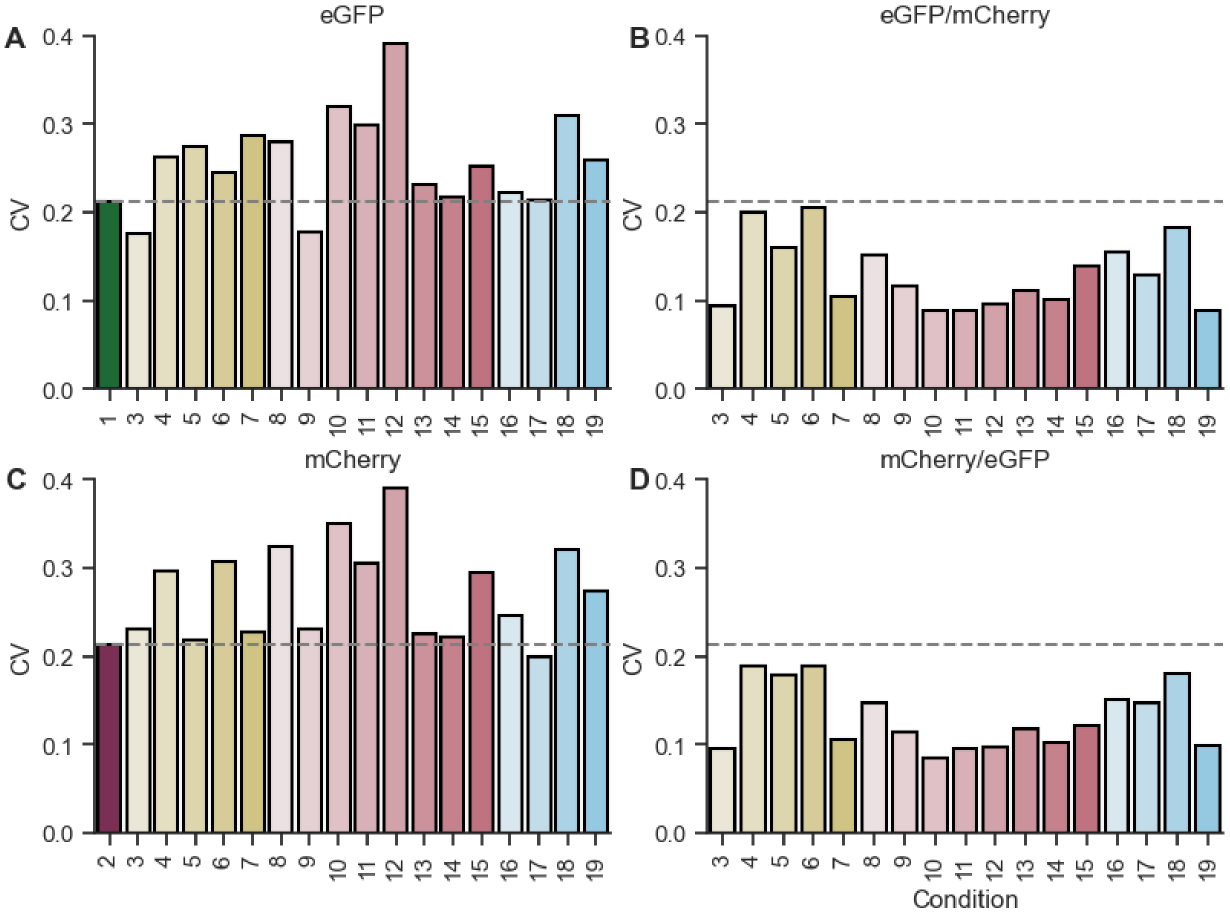

Supplementary Figure 11. Global CVs calculated from all discs from both experimental replicates, pooled, for **A)** eGFP, **B)** eGFP/mCherry, **C)** mCherry, and **D)** mCherry/eGFP of the normalization schemes from Fig. 2 (n=96 leaf discs). The dotted line in **A)** and **B)** is the eGFP CV of scheme 1, and the dotted line in **C)** and **D)** is the mCherry CV of scheme 2. Source data for this figure is available in the Source Data file.

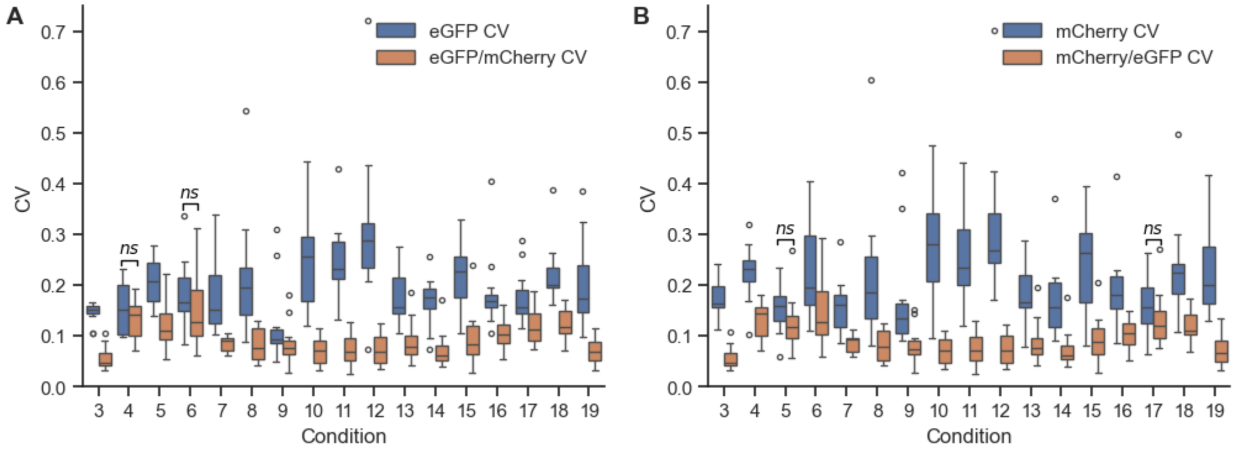

Supplementary Figure 12. Comparison of unnormalized CVs to normalized CVs from Fig. 2 when **A)** eGFP is the reporter and mCherry the normalizer or when **B)** mCherry is the reporter and eGFP the normalizer. A paired, one-tailed Student's *t*-test was conducted, where each pair was the reporter CV and the reporter/normalizer CV from the same plant. The CV for each individual plant is calculated from 8 leaf discs. For visual clarity, only conditions for which the reporter/normalizer CVs were not significantly lower than the reporter CVs ($p>0.05$) are marked. Otherwise the reporter/normalizer CVs were significantly lower ($p<0.05$). Boxes show the median and IQR, and whiskers show the minima and maxima, excluding outliers (beyond 1.5 IQR). Circles indicate outliers. Each boxplot represents n=48 discs. Source data for this figure is available in the Source Data file.

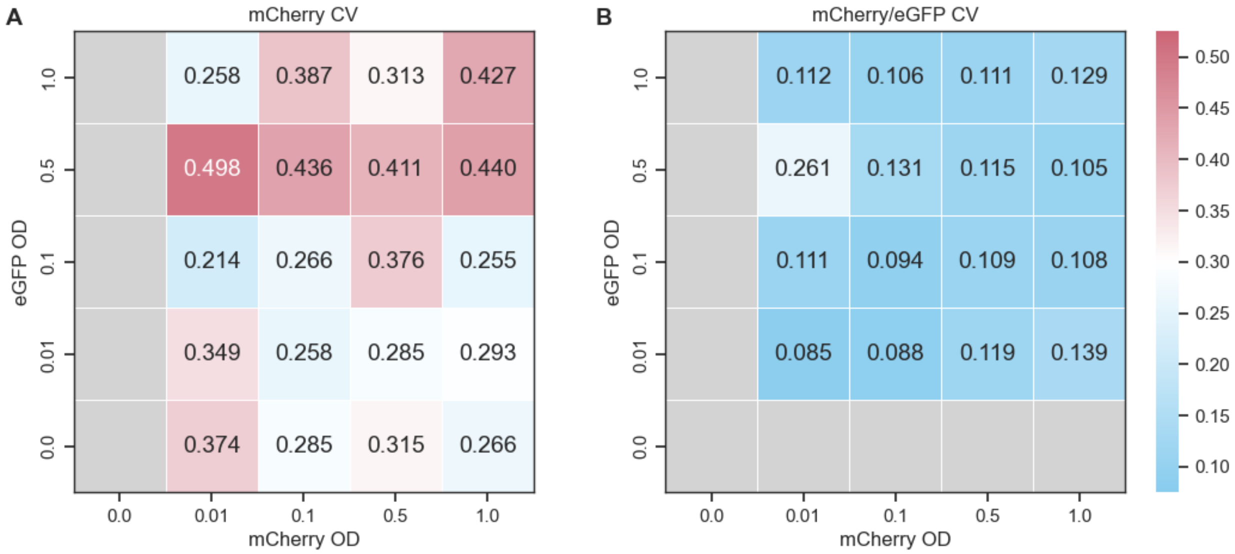

Supplementary Figure 13. Data from Fig. 3 showing mCherry as the reporter and eGFP as the normalizer instead. Matrices of all OD combinations' CV of **A)** the mCherry fluorescence and **B)** ratio of mCherry/eGFP. Six plants per OD combination, two leaves per plant, and four discs per leaf, for a total of n=48 discs. Source data for this figure is available in the Source Data file.

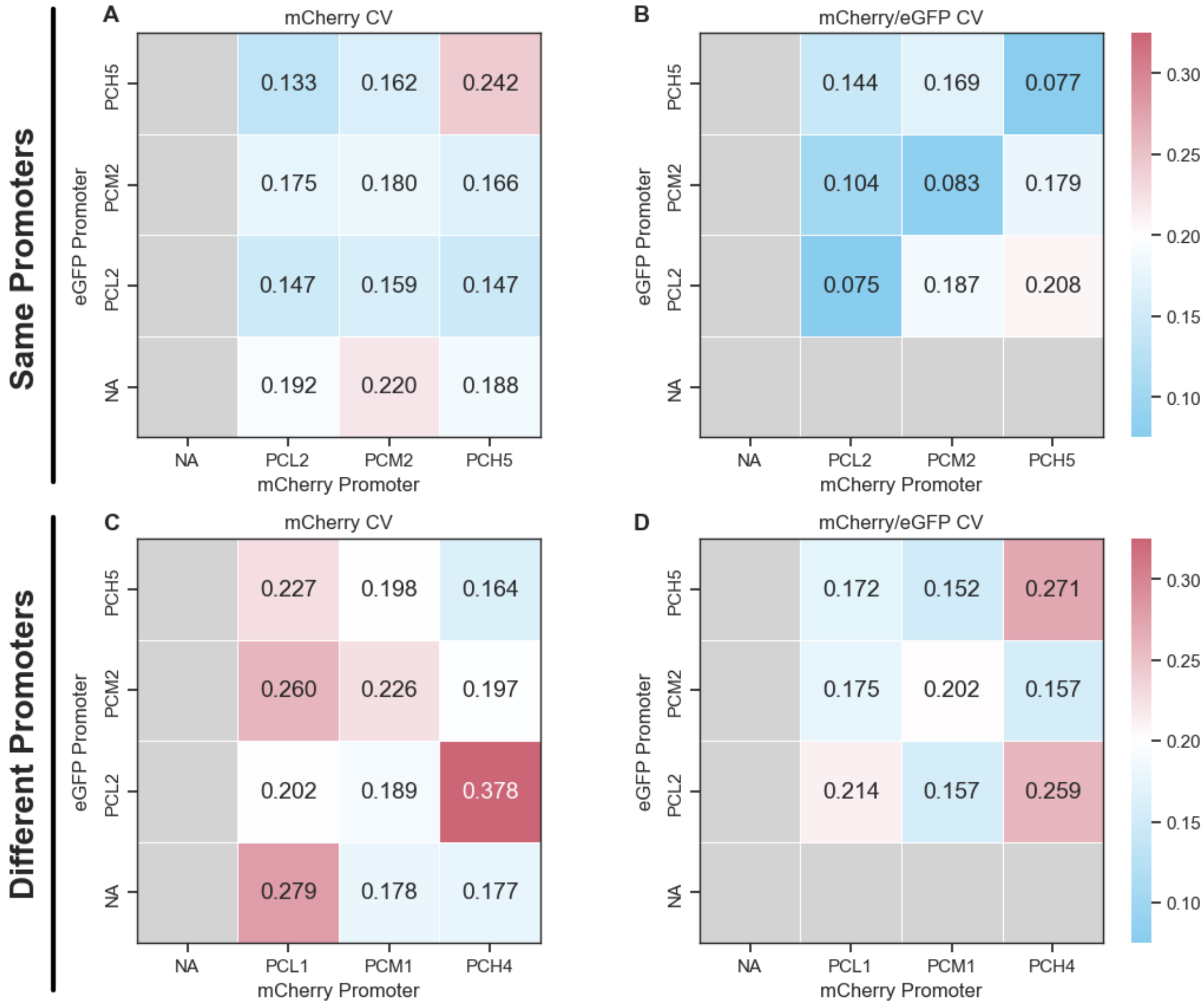

Supplementary Figure 14. Data from Fig. 4 showing mCherry as the reporter and eGFP as the normalizer instead. Matrices of all promoter combinations' CV of **A)** the mCherry fluorescence and **B)** ratio of mCherry/eGFP when the set of 3 promoters are the same for eGFP and mCherry binary vectors (PCL2, PCM2, PCH5). Matrices of all promoter combinations' CV of **C)** the mCherry fluorescence and **D)** ratio of mCherry/eGFP when the set of 3 promoters for mCherry binary vectors (PCL1, PCM1, PCH4) are different than the set for eGFP. Six plants per promoter combination, two leaves per plant, and four discs per leaf, for a total of n=48 discs. Source data for this figure is available in the Source Data file.

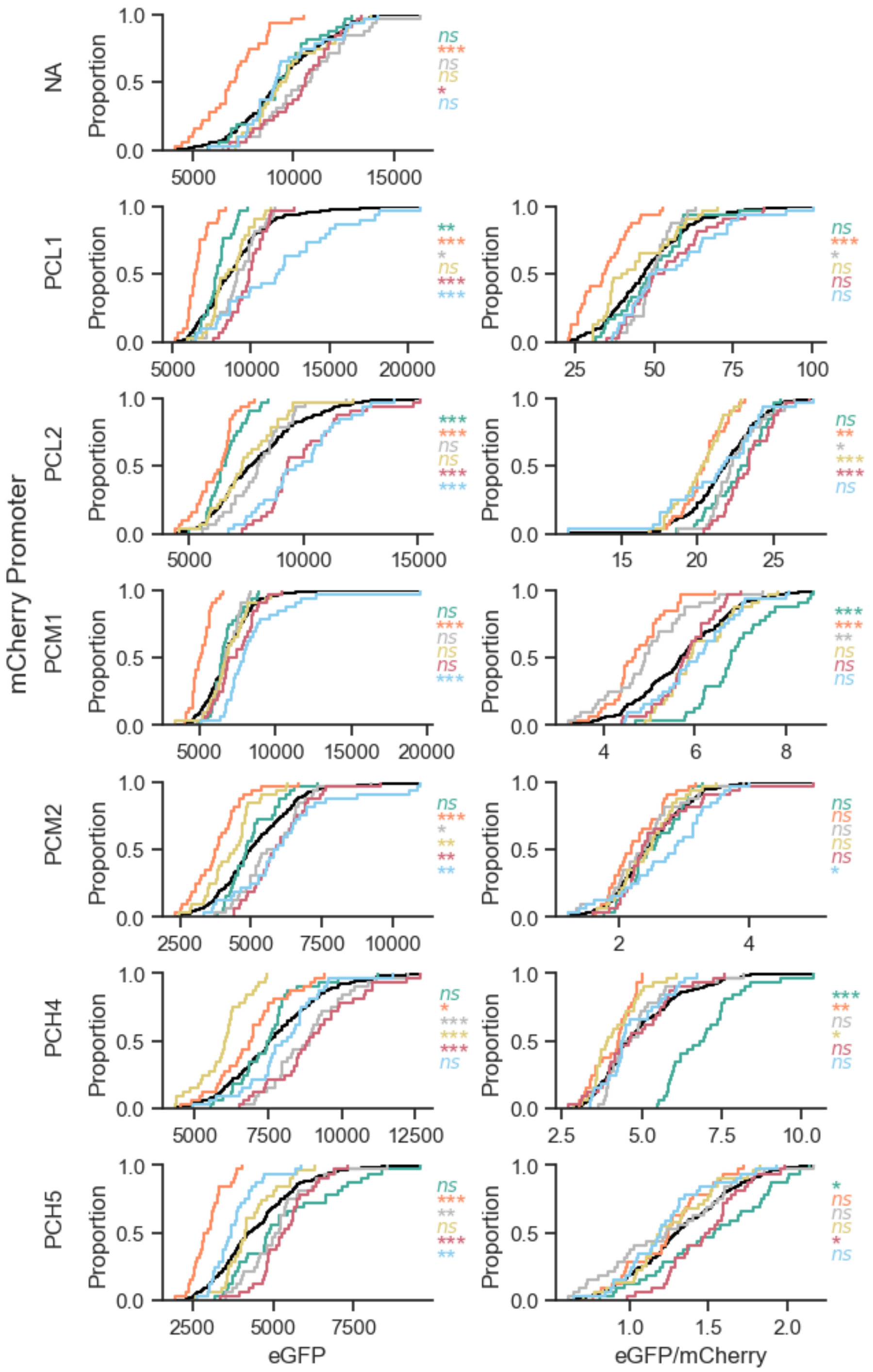

Supplementary Figure 15. Cumulative density functions of all conditions tested in Fig. 5: PCL2:eGFP alone or normalized by mCherry driven by PCL1, PCL2, PCM1, PCM2, PCH4, or PCH5. Left: eGFP, right: eGFP/mCherry. Each experimental replicate is a unique color. The black line is the CDF for the pooled data of all six experimental replicates. The p-values of one-sample Kolmogorov-Smirnov tests appear to the right of each CDF, colored by experimental replicate. Asterisks indicate p-values: * < 0.05, ** < 0.01, *** < 0.001, and ns = not significant. Four plants per promoter combination, two leaves per plant, 4 discs per leaf, for total of n=32 discs per

experimental replicate; 6 experimental replicates. Source data for this figure is available in the Source Data file.

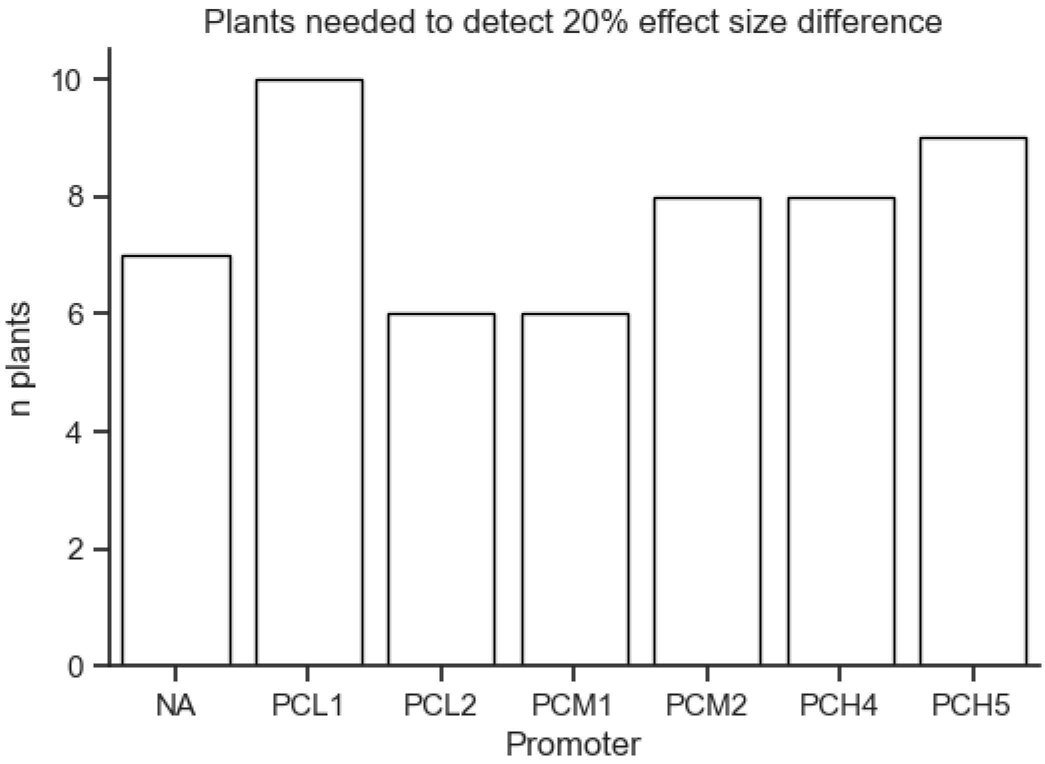

Supplementary Figure 16. Number of plants needed to detect a 20% effect size difference given the CVs of the conditions tested in Fig. 5. Source data for this figure is available in the Source Data file.

Supplementary Method 1

Full-length protocol

This protocol has been optimized for the JBEI plant growth room conditions (long day, ~120 ppfd, 23°C, 65% humidity). Plant needs may vary when humidity / temperature / light intensity / light cycle are altered. All plants cycle through dedicated 1 week-, 2 week-, 3 week-, and 4 week-old zones to minimize batch-to-batch variation.

Soil prep: Sungro Sunshine mix #4 (aggregate plus), supplement with Osmocote (14-14-14) pellets at 1.5 TBSP (~20 mL) / 4L. Mix in the osmocote well and manually break apart large chunks of soil. When preparing soil for seedlings, a layer of Pro-Mix PGX is added as topsoil. Plants are grown in Greenhouse Megastore 3.11" x 3.11" x 2.25" pots (Traditional Insert: CN-IKN-1801) which fit 18 pots per flat (1020 Trays Heavy Duty: CN-FLHD-X2).

If tasks are split between multiple individuals, we recommend that the same individual(s) do the same tasks each week to minimize batch-to-batch variation.

Day 0: Germinate seeds

- Fill an adequate number of pots with osmocote-supplemented soil (usually ~50-100 good seedlings per pot).
- Wet the soil by pouring excess tap water from above and allowing it to drain through
- Add a liberal layer of topsoil, and thoroughly wet it. Since topsoil is very fine and tends to be hydrophobic, this is easiest to do with a spray bottle.
- Using a spatula, sprinkle a pinch of seeds over the soil, ideally 50-100 seedlings per pot. If they germinate too densely, they will be smaller at transplant time. More sparse, healthy seedlings tend to grow into larger, healthy mature plants.
- Pour 0.5 L of tap water into the bottom of the tray to ensure high humidity.
- Put pots into a tray and cover with a hood (all vents closed) to keep humidity high
- Seedlings should be ready to transplant in one week

Day 7: Transplant seedlings

- Prep soil with osmocote as described above. For each flat to be transplanted, take a sheet of 18 pots, place it in the flat tray, and fill all to the brim. (Flats can be stacked to conserve space).
- Break out only the corner pot from each flat and add 3 L of tap water to the bottom of the tray.
- Allow the soil to soak for at least 60 minutes, until the top of the soil looks wet.
- After soaking, pour the excess water out from the bottom of each tray.
- Break apart the 18 pots in the flat. If the plants get big and the pots are still connected, it is very difficult to separate them without damaging the plants.
- Transplant seedlings.
    - Make a small hole in the soil of all the seedling pots.

- Gently remove seedlings from the germination pot and place them inside their own individual pot, root inside the hole.
- Once all the seedlings are placed in their own pot, push the soil compact around the roots to secure the seedlings in the soil.
- *Be careful not to damage the roots*. Any seedling with a snapped root will be stunted and may not survive transplantation; throw it away. Grip seedlings by one of the cotyledons (forceps may help) so as not to damage the stem/roots.

- Place a hood over each flat, *hood vents closed*, and move the flat into a growth room/chamber into the 1 week-old plant zone. The transplanted seedlings should require no attention for the next week.

Day 14: Open hood vents

- Move the post-transplantation flats from the 1 week-old plant zone to the 2 week-old plant zone. Open the top and both side vents on all of their hoods.
- This is important to allow the humidity within the hood to slowly equilibrate to the outside conditions. Removing the hood all at once causes a rapid humidity change which can be detrimental.

Day 19: Remove hoods

- Remove the hood from each flat.

Day 21: Water plants

- Move flats from the 2 week-old zone to the 3 week-old zone.
- Give each flat 1 L of tap water. It is easiest to pour the water into the bottom of the tray using a funnel.

Day 26: Separate plants into 9/flat and water

- Move the flats from the 3 week-old zone to the 4 week-old zone.
- Separate the 18 plants in each flat into two flats with 9 plants each, checkerboard pattern to maximize space for each plant.
- Checkerboarding helps prevent overcrowding, shade avoidance, and loss of structural integrity.
- Give each flat 1 L of tap water. Pour directly into the bottom of the tray.

Day 29: Agroinfiltration

- Infiltrate plants as desired.
- Post-infiltration, give each tray of plants 1 L of tap water so they are not dried out 72 hours later.

Quick guide

Tuesday

- Remove the hoods from flats in the 2 week-old zone.
- Separate flats in the 3 week-old zone into 9 plants/flat (checkerboarded). Move into the 4 week-old zone and give each flat 1 L of tap water.

Thursday

- Prepare soil for as many flats as desired and saturate each in 3 L water/flat for at least 1h.
- Move the flats in the 2 week-old zone to the 3 week-old zone. Add 1 L of tap water/flat.
- Move the flats in the 1 week-old zone to the 2 week-old zone. Open all vents on the hoods of these flats.
- Drain the water from the flats for transplantation and break apart the pots.
- Transplant one seedling to each pot, cover with a hood (all vents closed), and place in the 1 week-old zone.
- Sow seeds for next week.

Friday

- Infiltrate.
- Recover each flat of infiltrated plants in the 4 week-old zone with 1 L of tap water/flat.

Example calendar

Letters represent unique plant batch IDs. IDs are in alphabetical order from oldest to youngest.

Wk	Mon	Tue	Wed	Thu	Fri
1				Sow seeds (A)	
2				Sow seeds (B)<br>Transplant seedlings (A)	
3				Sow seeds (C)<br>Transplant seedlings (B)<br>Open vents (A)	
4		Remove hoods (A)		Sow seeds (D)<br>Transplant seedlings (C)<br>Open vents (B)<br>Water (A)	
5		Remove hoods (B)<br>Separate pots, water (A)		Sow seeds (E)<br>Transplant seedlings (D)<br>Open vents (C)<br>Water (B)	Infiltrate, water (A)
et cetera					

Supplementary Method 2

Monte Carlo Simulation

A dataset of 1,813 *N. benthamiana* plants from 32 independent GFP transient expression experiments was compiled, spanning multiple years, researchers, and binary vector designs using either *A. tumefaciens* GV3101 (1,087 plants) or EHA105 (726 plants). This dataset captures the real-world variability of transient expression performance outside of a single controlled experimental environment.

For each plant, a coefficient of variation (CV) of GFP expression was calculated from eight leaf disks. Using these data, we parameterized a hierarchical Monte Carlo model to reproduce observed variability at three levels: (i) week-to-week shifts in average plant CV, representing overall plant quality between experiments; (ii) plant-to-plant heterogeneity within a given week; and (iii) within-plant disk-level noise.

Weekly median plant CVs followed a log-normal distribution with parameters meanlog = -1.447, sdlog = 0.300 for GV3101 and meanlog = -0.903, sdlog = 0.460 for EHA105. The within-week spread of plant CVs (weekly SD-of-CV) also followed a log-normal distribution (meanlog = -2.3248, sdlog = 0.3897). Fit adequacy was confirmed using Kolmogorov–Smirnov and density RMSE metrics.

These data were used to simulate variance across random weeks. For each simulated experiment, a week quality value was selected using a random number from the log-normal distribution of the strain of choice (GV3101 vs EHA105), representing the average plant CV for that week. Additionally, a week quality spread value—modeled from the empirical standard deviation of plant CVs across different experiments—was randomly selected from the underlying log-normal distribution and used for the standard deviation of CV values for that week. These two values were then used to generate individual plant CVs for the simulated experiment, incorporating both the mean and standard deviation values for plant CV that were randomly generated for that week. Each plant drawn from this distribution thus has its own unique CV value, bounded by biologically relevant values between 0.03-2.0, which were inferred from the 1813 measured plant spread.

For each simulated plant, 8 leaf discs were generated using an arbitrary mean value of 100,000 GFP units and standard deviation derived from the plant CV. A given simulated experiment would then have all leaf disc data bulked (e.g. 10 plants with 80 total discs) and used for population-level comparisons. For the modeling in this study to determine features such as minimal detectable effect size or power analyses, 1000 weeks of experiments were simulated using the above pipeline. Simulation outputs were summarized as statistical power (fraction of iterations with significant construct differences) across plant counts and effect sizes. Comparison of features such as effect size was conducted through two-sided t-tests with Benjamini–Hochberg FDR control at $\alpha=0.05$. For power analysis modeling, a ≥95% accurate detection of differences was used as a threshold for classifying sufficient detection power.

To validate the outputs of this simulation, goodness-of-fit between simulated and empirical CV distributions was assessed using KS and 1D Wasserstein distances (Fig. 6b).

Mixed effect modeling

Modeling of variance was conducted using the pCM2::GFP dataset which consisted of GFP expression data from 114 plants collected over 15 weeks. Fluorescence data (n = 8 discs per plant) were transformed to a log10 scale to linearize multiplicative effects and to account for heteroscedasticity.

On this scale, a linear mixed-effects model was fit to account for 4 major sources of variation: (i) experiment-to-experiment shifts in plant mean, (ii) experiment-to-experiment shifts in intraplant variance, (iii) plant-level shifts in mean GFP expression, and (iv) leaf-to-leaf expression variability, along with a residual component. The equation for modeling such variance for the GFP expression of a given leaf disc is as follows:

$Log_{10}GFP_{(disc,\ plant,\ week)}$ =
β0 (dataset mean) +
β1 * Leaf position (dataset difference between top and bottom leaf) +
u_0 (week's deviation in mean GFP) +
u_1 * Leaf position (weeks deviation in top vs bottom leaf difference) +
v_0 (Individual plant's mean deviation) +
v_1 (Individual plant's leaf-to-leaf variation) +
e (residual error)

This was calculated using the R code:

```
m_final <- lmer(
log_GFP ~ Leaf_c +
(1 + Leaf_c | Date) +
(1 + Leaf_c | Unique_plant_ID),
data = PC4_data, REML = TRUE)
```

In the above, Leaf_c encoded the contrast between the top and bottom leaves.

Disc-to-disc, positional, and technical variance:

To determine the impact of disc-to-disc variability, leaf position, and technical replication noise on total observed variability, the same pCM2::GFP construct was used to infiltrate 24 plants on the same two leaves (T4 and T5) in two positions (proximal to the petiole and distal) with two *Agrobacterium* strains (GV3101 and EHA105, 12 plants per strain). For each leaf, eight discs were taken (four per proximal or distal position). GFP fluorescence was quantified as before, except plates were scanned three times each in their standard position and then were flipped 180 degrees and scanned three more times to enable quantification of technical variance. The

following R code when then used to fit a mixed effects model to better account for residual variation:

```
m_leafc <- lmer(
log_GFP ~
Plate_Position + (location of disc on the plate) +
Leaf_c + (Leaf T4 or T5 variance)
(1 + Leaf_c | Plant) + (Plant mean and leaf-to-leaf variance)
(1 | Plant:Leaf:Site) + (Proximal vs distal position impact)
(1 | DiskID) + (disc-to-disc variation),
data = punchvar, REML = TRUE)
```

Ruby and PDC modeling

Modeling of metabolite production variance was conducted in a similar way but with reduced variables given tissue bulking that was done prior to extraction. For Ruby, a single infiltrated spot per leaf was excised and extracted, resulting in data for only week-to-week and plant-to-plant variability. As only a single measurement was taken per leaf, the residual of this model includes leaf-to-leaf variability, as calculated by the following R code:

```
m_ruby <- lmer(
log10_ruby ~ 1+
(1 | Experimental_replicate) + (experiment mean variation)
(1 | Experimental_replicate:Plant), (plant level variation within experiment)
REML = TRUE)
```

For PDC production, tissue from both leaves was bulked prior to extraction. For this reason, a linear model was used to examine the relationship between week and PDC production, with residual variance representing plant-to-plant and leaf-to-leaf variability not explained by week-to-week differences.
